# Using Phage and Yeast Display to Select Hundreds of Monoclonal Antibodies: Application to Antigen 85, a Tuberculosis Biomarker

**DOI:** 10.1371/journal.pone.0049535

**Published:** 2012-11-14

**Authors:** Fortunato Ferrara, Leslie A. Naranjo, Sandeep Kumar, Tiziano Gaiotto, Harshini Mukundan, Basil Swanson, Andrew R. M. Bradbury

**Affiliations:** 1 Bioscience Division, Los Alamos National Laboratory, Los Alamos, New Mexico, United States of America; 2 Chemistry Division, Los Alamos National Laboratory, Los Alamos, New Mexico, United States of America; National Jewish Health and University of Colorado School of Medicine, United States of America

## Abstract

**Background:**

Current diagnostic methods for tuberculosis (TB), a major global health challenge that kills nearly two million people annually, are time-consuming and inadequate. During infection a number of bacterial molecules that play a role in the infective process are released and have been proposed as biomarkers for early TB diagnosis. Antigen 85 (Ag85) is the most abundant secreted TB protein, and a potential target for this diagnostic approach. One of the bottlenecks in the direct detection of such bacterial targets is the availability of robust, sensitive, specific antibodies.

**Methods:**

Using Ag85 as a model, we describe a method to select antibodies against any potential target using a novel combination of phage and yeast display that exploits the advantage of each approach.

**Results:**

The efficiency of this approach was attested to by the 111 specific antibodies identified in initial screens. These were assessed for binding to the different Ag85 subunits, affinity, and activity in sandwich assays.

**Conclusions:**

The novelty of this approach lies in the possibility of screening the entire output of a phage antibody selection in a single experiment by yeast display. This can be considered analogous to carrying out a million ELISAs. The monoclonal antibodies (mAbs) identified in this way show high binding affinity and selectivity for the antigens and offer an advantage over traditional mAbs produced by relatively expensive and time consuming techniques. This approach has wide applicability, and the affinity of selected antibodies can be significantly improved, if required.

## Introduction

*Mycobacterium tuberculosis*, the causative agent of tuberculosis (TB), is a global health problem with 2–3 millions deaths and 8 million active infections annually [1]. Inappropriate treatment and other factors has led to the development of multi-, extensively- and totally drug resistant *M. tuberculosis* strains [2], some of which have recently been shown to have enhanced person to person transmission [3].

Efficient TB diagnosis and treatment are crucial for control, with early diagnosis allowing rapid treatment and reduced spread. The standard screening method is the Tuberculin Skin Test, which lacks sensitivity and specificity, making it less useful for people at low risk [4]. The gold diagnostic standard remains clinical examination with sputum examination and cultures for acid-fast bacilli. However, these require expertise, and are not sensitive enough to detect more than 65–70% of cases. Other techniques such as BACTEC, fluorescent antibody tests, gas chromatography, DNA hybridization, PCR and radioimmunoassays are sensitive, but require established laboratories [5].

A straightforward, rapid, sensitive test would significantly alleviate the burden of this disease: mathematical modeling indicates that an “ideal” diagnostic assay with 100% sensitivity, specificity and access could prevent 36% of all TB related deaths [6]. Enzyme-linked immunosorbant assays (ELISAs) are relatively simple and may satisfy many “ideal’ TB assay characteristics. Two ELISA approaches have been taken to diagnose TB. In the first, the response to infection is detected by assessing recognition of different TB antigens by host antibodies [7], [8]. These have relatively high sensitivity (>80%) and specificity (>80%), and can detect TB specific antibodies in smear and culture negative patients. Greater sensitivity and specificity have been obtained in microarrays using 17 [9] or 54 different recombinant TB antigens [10]. A recent meta-analysis [11] concluded that assays based on multiple antigens provided higher sensitivities and specificities than single antigen assays, with 38 kDa, Ag85B, a-crystallin and MPT51 being most effective [12]. An intrinsic problem with this approach is the variability in individual immune responses, differences in disease stages, and the time taken to develop an antibody response. Furthermore, antibody presence is not always indicative of an active TB infection. The second approach involves direct detection of the antigens themselves, rather than the antibodies that recognize them. Many have been assessed [13], with the detection of antigen 85 (Ag85) components directly in serum [14], [15] being particularly diagnostic.

Ag85 comprises three related (71–77% homology) proteins of 30–32 kDa each: Ag85A, Ag85B, Ag85C [16], that are the most abundantly secreted *M. tuberculosis* proteins in vitro [17]. Ag85 and Ag85 RNA have been explored as potential biomarkers for TB in urine [18] and sera [14], with high sensitivity and specificity using indirect ELISA. Using commercially available antibodies on a waveguide biosensor platform we obtained a 500 fM limit of detection for Ag85. However, the application of this assay to clinical samples was prevented by poor antibody stability (Mukundan H. et al, Tuberculosis, in press, 2012), prompting the present study to develop antibodies for this significant biomarker.

**Figure 1:**
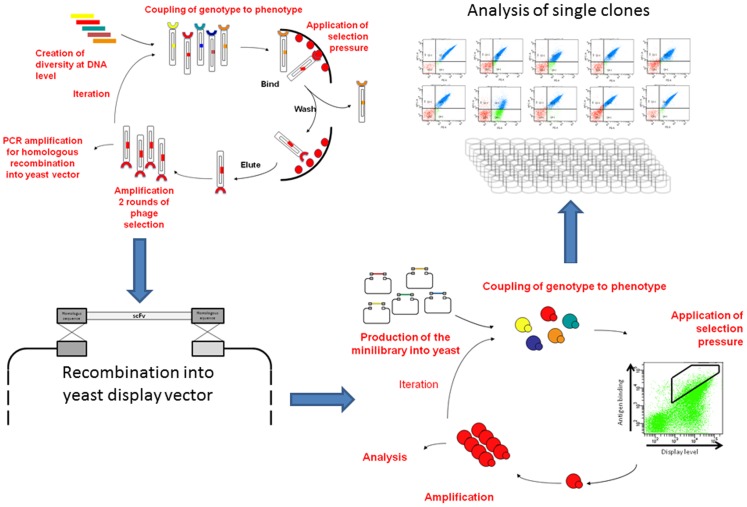
Strategy applied to the selection of antibodies using both phage and yeast display. Antibodies are first selected against Ag85 using two rounds of phage display, after which the whole selection output is cloned into a yeast display vector using homologous recombination. A further one or two rounds of sorting by flow cytometry allow the subsequent isolation and testing of single clones.

Immunological reagents used for infectious diagnosis typically comprise poly- or monoclonal antibodies from immunized animals. Recently antibodies have been developed using *in vitro* methods [19], [20]. In phage display, antibodies of interest can be selected from large phage antibody libraries in only a few days, rather than the months required for immunization approaches. Yeast display has recently emerged as an alternative strategy, with one significant advantage over phage display: the ability to accurately control selection parameters by flow cytometry [21].

**Figure 2:**
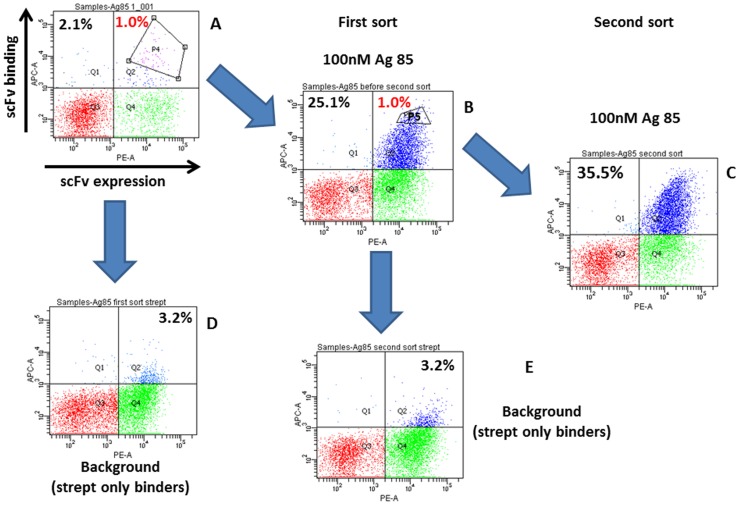
Yeast cell analysis before and after sorting. 2A shows the yeast population after cloning the Ag85 selected phage display output. The upper right quadrant (Q2) representing those yeast that are displaying scFv and binding antigen contains 2.1% of all yeast cells, of which the top 1%, as indicated by the sort gate, were sorted. 2B shows the outcome of the first sort after yeast were collected and grown up. Q2 now contains 25.1% of all yeast cells, of which the top 1% were again sorted as shown. 2C shows the outcome of the second sort: Q2 now contains 35.5% of all yeast cells. 2D and 2E show the background streptavidin binding clones that represent, for both the first and the second sort, 3.2% of the total population.

In the present work, we take advantage of both selection approaches to isolate numerous single chain Fvs (scFvs) recognizing Ag85. We first carried out a selection against Ag85 from a large naïve phage scFv library [22], and after this “enrichment” step, the selection output was cloned into a yeast display system. A large number of different highly specific Ag85 antibodies was isolated, confirming the utility of the combination of these two in vitro methods for the production of “tools” for a precise a reliable diagnosis of TB infection.

## Materials and Methods

### Strains

DH5αF′: F′/endA1 hsdR17(rK−mK+) supE44 thi-1 recA1 gyrA (Na1r) relA1 D(lacZYAargF) U169(m80lacZDM15) was used for phage display and sequencing analysis.

**Figure 3:**
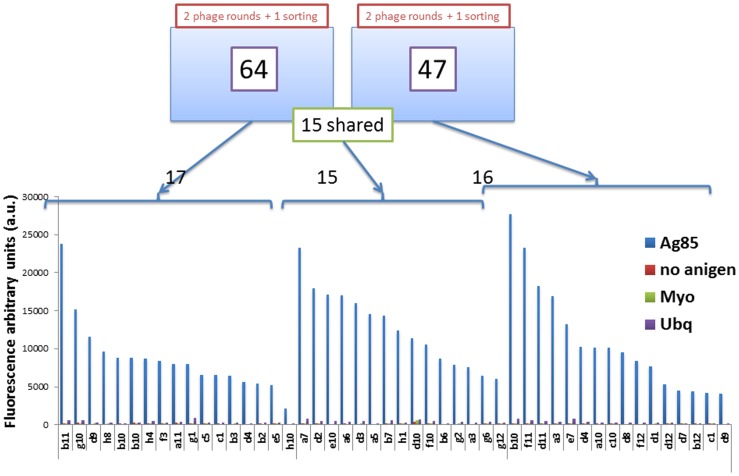
Specificity of selected monoclonals. 48 different monoclonals chosen from among the 111 identified were tested for specific binding to Ag85, as well as two irrelevant antigens (myoglobin and ubiquitin). All were positive for Ag85, showing signal to noise ratios exceeding 8.

**Table 1:** CDR3 protein sequences of the different scFvs obtain after one or two round of yeast display sorting.

CDR3 2 phage rounds +1 sorting	aa	CDR3 shared clones	aa	CDR3 2 phage rounds +2 sorting	aa
CARGGYPVHYWGQ	13	CAKAQTTRRSIWGQ	14	CARGFTGDYWGQ	12
CATGRAYYFDSWGQ	14	CVRRGRSLADVWGQ	14	CARIRSGYPDYWGQ	14
CARAKGYWYFDLWGR	15	CAKRPWGPYWYFGLWGR	17	CARVLPGPYQYWGR	14
CARFIRGVMVDYWGQ	15	CAKAKSYYDSSGYYPWGQ	18	CVGTSSVWYFDLWGR	15
CVRKSSYGVFDIWGQ	15	CAKVRGSYRSRYFDLWGR	18	CAKGTAVVRAFDLWGR	16
TAVYYCSNGGLVWGQ	15	CARRGRHLGVRAFDIWGQ	18	CAKVQWLRPRFDYWGQ	16
CAKWGGRNGAFDIWGQ	16	CARVPAGPPYWYFNLWGR	18	CARIRYRTGGIDYWGQ	16
CARDYYGQAYFQHWGQ	16	CARGGLHPYSYYGMDVWGQ	19	CARVWGSGWYPPHWGQ	16
CARVGPMYWYFDLWGR	16	CARAVVRSGWRPWYFDLWGR	20	CARHFSITTQWGMDVWGQ	18
CVRRSISTGAFDIWGQ	16	CARFRISRGYYYYGMDVWGQ	20	CAKLLAWTTAKRYFDLWGR	19
CAKLGRIAAAGKVHWGQ	17	CARRLRIAVDHRYGFDIWGQ	20	CAKLPSSYYYYYGMDVWGQ	19
CARDSYSSGWPIDYWGQ	17	CARVGSSWPYYYYGMDVWGQ	20	CAKQRSTASYYYGLDVWGQ	19
CARKPSNSYSWFGPWGQ	17	CARVPGGSYYPYYYMDVWGK	20	CARAGDYVWGSYRLAYWGQ	19
CARQLMVRGNRYDYWGQ	17	CARRGAAMGNYYYYGMDVWGR	21	CARDRIAARPRWYFDLWGR	19
CAVRRRGVINGMDVWGQ	17	TAVYYCGKGMVPYPHGGPWGQ	21	CAGRKPYSSSWYKAGDYWGQ	20
CVRGWGAYKGNFDYWGQ	17	CARHRVSWAIRTNSKYGMDVWGQ	23	CAKFGSGWYSGHYGMDVWGQ	20
CARGRSRWPPWFFDYWGQ	18			CARADSGWYGGRRGFDPWGQ	20
CARRRYSSSSNGFDIWGQ	18			CARGRYDPYYYYYGMGVWGQ	20
CARTRFIRGLRYFDYWGR	18			CARIPGGSSSWYMKFDPWGQ	20
CASDKSGSYSGGGGYWGQ	18			CASPLLRFLPGYYGMDVWGQ	20
CARDSLSSLASDAFDIWGQ	19			CAKVGRGSGWSPYWYFDLWGR	21
CARGRRVTMVRGVVNYRGQ	19			CARGGRYPRHYYYYGMDVWGQ	21
CARITSYNGNHYGMDVWGQ	19			CARGRRWPPSPGNKAFDYWGQ	21
CARRKGGSSRGWYFDLWGR	19			CASGYDFWSGYPYWYFDLWGR	21
CARTVKGDYPYWYFDLWGR	19			CARDLSSYGQRLYGDYGWYWGQ	22
CAKLGPIPRSRYWYFDLWGR	20			CARGKGSYSSRPTPRYFDLWGR	22
CARAFLPYSSGWWHYFDYWGQ	21			CARGVVVPAAVYYYYGIDVWGQ	22
CARDSRFGSGSYSTRFDYWGQ	21			CARGYSSSWIGFYYYYYMDVWGK	23
CARERRTRGYSYGRLLDYWGQ	21			CAREHTAMVSGRVPYYYYMDVWGK	24
CARWSRWGRSYAVNWFDPWGQ	21			CARILRGVTNYGGRRYYYGMDVWGQ	25
CTRWSSGSYYYYYYGMDVWGQ	21			CARDPYYDFWSGYLNYYYYGMDVWGQ	26
CARDRYDFLPNLYYYYMDVWGK	22			CARGFGGYCSGGRCRYRGYYYYMDVWGK	28
CARLSRGGRFVFYYYYMDVRGK	22			CARYSRTDRRKAGGYDSSGYMKTDYYYYGMDVWGQ	35
CARQVHSSGWYYYYYGMDVWGQ	22				
CARDKYYGSGSYNYYYYMDVRGK	23				
CARWSYIWGSYRPRYWYFDLWGR	23				
CARIYAPLYYYGSGSLYYSDYWGQ	24				
CARLGGRGPAIAARQVWYFDLWGR	24				
CARVPLRGYSGYDYIRQKFDIWGQ	24				
CARVTYYDFWSGYSLGGHFNLWGR	24				
CAKSPRRYFDGRYYYYYYGMDVWGQ	25				
CAREKGILKGAPYYYYYYGMDVWGQ	25				
CARSGYSSSWYNPYYYYYGMDVWGQ	25				
CARTPYYYGSGSYYNALRYFDLRGR	25				
CARDPWYSYGQHLHDYYYYGMDVWGQ	26				
CATTSGYDFWSGYYPYYYYYGMDVWGQ	27				
CARHGTTSYDFWSGFRKYYYYGMDVWGQ	28				
TAVYYCTTDWGPAWGSYRWYYYGMDVWGQ	29				

*S. cerevisiae* EBY100 (GAL1-AGA1::URA3 ura3-52 trp1 leu21 his3200 pep4::HIS2 prb11.6R can1 GAL) was used for yeast display [23].

**Figure 4:**
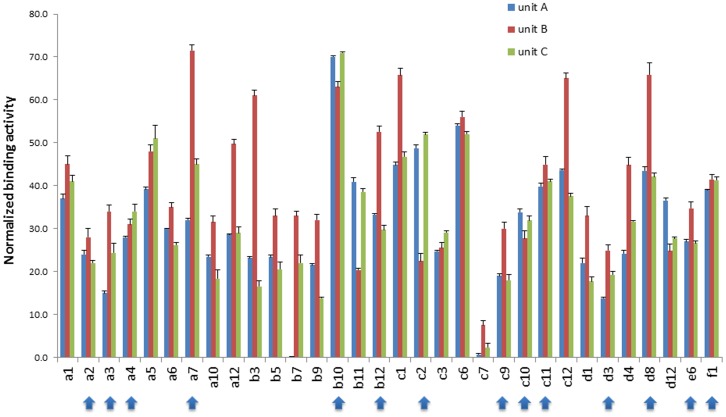
Binding of selected monoclonals to different Ag85 subunits. Each of the individual antibodies was tested for binding to the complete Ag85 complex, and the values obtained used to normalize the recognition of the 48 different scFvs for the three different Ag85 components. The arrowed clones were taken for further testing.

**Table 2:** Affinities of 14 antibodies determined directly on the surface of yeast.

	Kd scFv anti-Ag85 (nM)	group
A2	39	1 sort
A3	99.5	1 sort
A4	151	1 sort
A7	432	1 sort
avg 1 sort	180.375	
B10	98	com scFv
B12	110	com scFv
C2	97	com scFv
E6	22.1	com scFv
avg com scFv	81.775	
C9	160	2 sort
C10	59.9	2 sort
C11	120	2 sort
D3	84.5	2 sort
D8	202	2 sort
F1	96	2 sort
avg 2 sort	120.4	
total average	126.1	

Antibodies are identified as whether they were isolated after one yeast sort (1 sort), two yeast sorts (2 sort), or were found in common to both yeast sorts (com scFv).

### Antigen Preparation

The purified Ag85 complex and Ag85A, Ag85B and Ag85C components were obtained from BEI Resources, the purity of which was checked by SDS-PAGE. Purified Ag85 proteins were biotinylated using the Lightning-Link® Biotin kit (Innova Bioscience) following manufacturer’s instructions.

**Figure 5:**
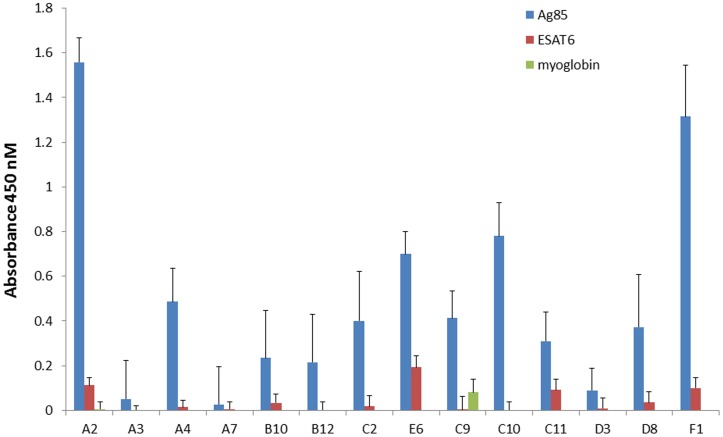
Enzyme linked immunosorbant assay (ELISA) of selected antibodies. 14 of the scFvs were recloned into a yeast expression vector as scFv-Fc fusions [28], using a rabbit Fc domain, and tested for recognition of Ag85 directly adsorbed to plastic in an ELISA format using standard anti-rabbit antibodies as secondary reagents.

### Phage Display Selection of scFv Antibodies

Our previously described naïve phage antibody library [22] was used to select Ag85 antibodies using an automated Kingfisher magnetic bead system (Thermo Lab Systems), allowing selection to be carried out in solution as previously described [24]. 0.5 µg of biotinylated Ag85 were used in the first round and 0.05 µg in the second. Capture of phage binding to biotinylated Ag85 was carried out using 2×10^7^ streptavidin magnetic beads (Dynabeads M-280).

**Figure 6:**
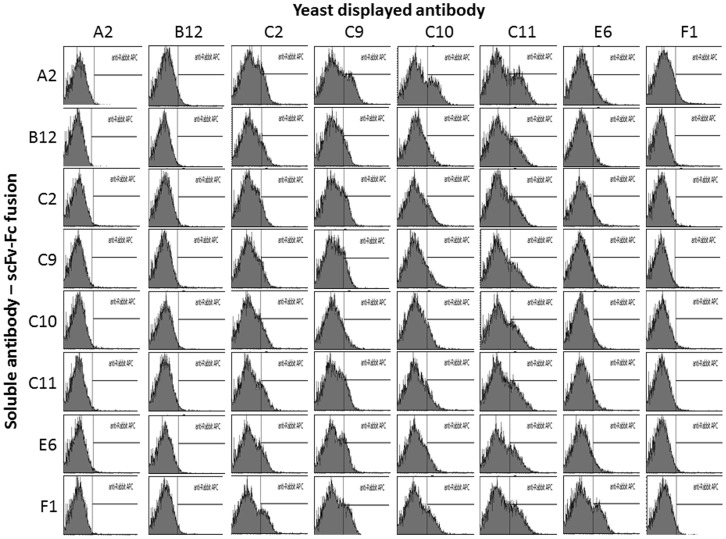
Sandwich ELISA of selected antibodies. Yeast displaying eight selected antibodies were used as Ag85 capture reagents, and tested with the same eight antibodies expressed as scFv-Fc fusions detected using fluorescent anti-rabbit secondary antibodies. Individual histograms show binding of the designated scFv-Fc fusion antibodies to individual yeast cells displaying the indicated antibodies in the presence of 100 nM Ag85. Fluorescence (i.e. the amount of scFv-Fc bound per individual yeast cell) is shown on the X axis, while the yeast count at each fluorescence level is shown on the Y axis. Yeast to the right of the vertical line in each plot are fluorescent as a result of a positive sandwich assay.

**Figure 7:**
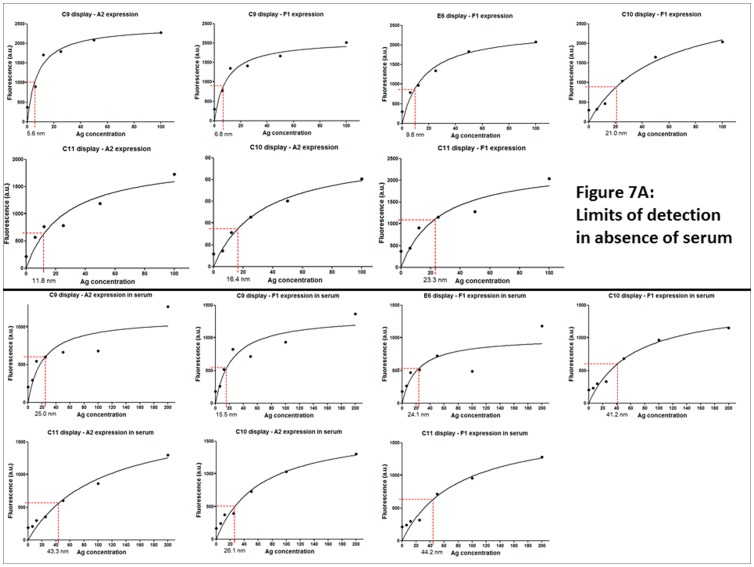
Ag85 detection limits in a sandwich assay. 10^6^ yeast displaying the indicated antibodies were induced, washed and resuspended in serial antigen dilutions. Binding was detected by flow cytometry following the addition of the indicated scFv-Fc fusion antibodies as induced culture supernatants, and fluorescent anti-rabbit secondary reagents. The indicated detection limits are defined as the minimal antigen concentration giving a signal three times greater than background. A: Detection in phosphate buffered saline. B: Detection in 1∶50 human serum diluted in PBS.

### Yeast Display of Anti-Ag85 scFvs

After two phage selection rounds DNA encoding the selected scFv antibodies was prepared using the QIAprep spin miniprep kit (Qiagen) and used as a PCR template for amplification with pDan5topDNL6-5′ (TCTGTGTGGTGGTGCGGCGCGCATGCC) and pDan5topDNL6-3′ (ATCCAGGCCCAGCAGT-GGGTTTGGGATTGGTTTGCC). The PCR product is compatible with our yeast display vector pDNL6 (a pPNL6 [20] derivative compatible with pDAN5 [22]), allowing cloning by gap repair [21].

**Figure 8:**
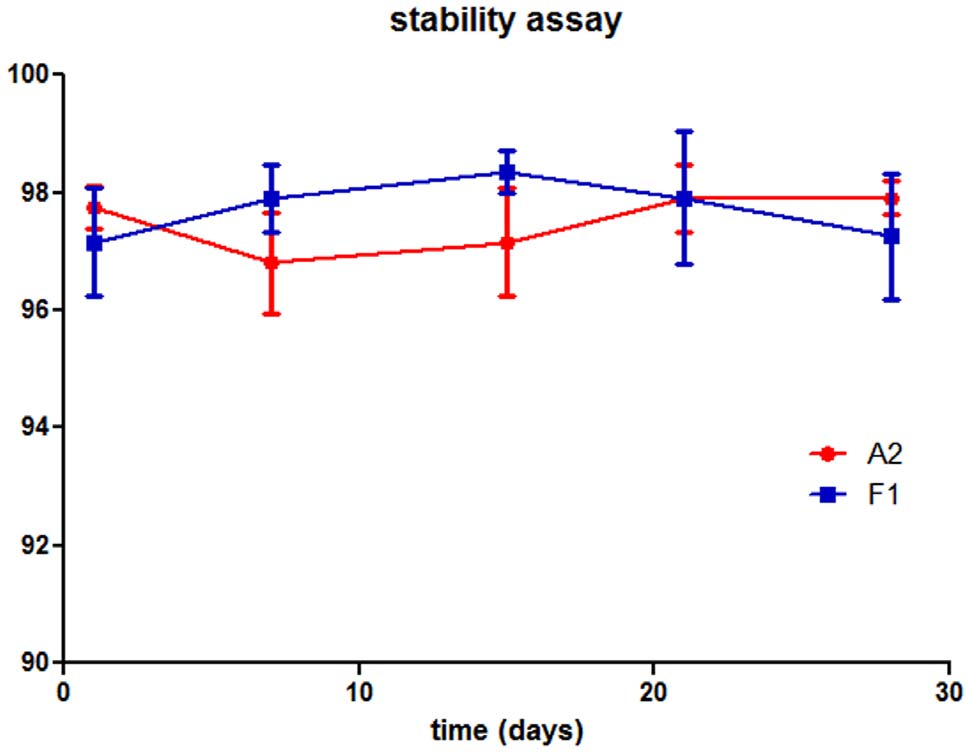
Stability test of yeast expressed scFv-Fc fusions. Unpurified scFv-Fc fusions were tested for binding to Ag85 by ELISA (Ag85 was adsorbed to plastic) at regular periods over 4 weeks. Results are expressed as percent signals obtained on the first day.

The yeast mini-libraries were further enriched by one or two rounds of sorting using flow cytometry using published methods [21]. Briefly, 2×10^6^ yeast cells were washed and resuspended in 100 µl of wash buffer containing 100 nM of biotinylated Ag85. Cells were labeled with streptavidin-AlexaFluor-633 to detect binding of biotinylated Ag85 and 1 µg of anti-SV5-PE to assess scFv display levels. Flow cytometry was performed using the FACSAria (Becton Dickinson), with yeast showing Ag85 and SV5 binding sorted. Collected cells were grown overnight at 30°C and induced for the next round of sorting. The sequences of sorted antibodies were determined on plasmid DNA prepared in DH5αF’ after transformation and clones were grouped based on the analysis of the CDRH3 region.

111 different scFvs were obtained. 48 of these were induced and tested for specific binding by flow cytometry (LSRII, Becton Dickinson) using biotinylated Ag85; myoglobin and streptavidin-Alexa-Fluor-633 were used as negative controls. The 48 clones were also tested for their binding activity for the different subunits (A, B and C) forming the Ag85 complex. The experiment was done in duplicate and standard deviation was calculated. BDiva software was used for flow cytometric analysis. The affinities of fourteen of the 48 clones were calculated directly on the yeast surface, which shows excellent agreement with other methods [25]. Approximately 10^6^ induced yeast cells were incubated with a dilution series of Ag85 complex (0–200 nM) detected as described above. The LSRII was used for flow cytometric analysis and BDiva software used for analysis. Quantitative equilibrium constants were calculated by plotting the streptavidin-AlexaFluor-633 normalized (APC antigen binding signal) mean fluorescence intensity against antigen concentration and fitting the curve using nonlinear regression analysis.

### Secretion and Purification of Soluble scFv-Fc Fusions and their Use in ELISA

scFv were subcloned into a modified pPNL9 vector [26] which expresses scFv-Fc fusions with rabbit Fc [Gaiotto et al. in preparation], with an SV5 tag at the C-terminus, allowing detection with anti-SV5 or anti-rabbit secondary reagents. After transformation into YVH10 [27], culture supernatant was used directly as a reagent after growth for 24–48 hours at 20°C. Binding of the scFv-Fc fusions was assessed by ELISA, using antigens - Ag85, ESAT6 (a different soluble protein produced by *M. tuberculosis*) and myoglobin - directly coupled to Nunc immunosorb microtiter plates, and 1/5 diluted yeast expression culture. Binding was detected with anti-SV5 HRP (horseradish peroxidase) or anti-rabbit-HRP. The produced scFv-Fc fusions were also tested for their stability over time. The concentration of recombinant antibodies was calculated at 1 mg/L and 100 µl of this solution, after different periods of storage, were used in ELISA assays as described above. A commercial rabbit monoclonal antibody (AbCam) at the same concentration was used as a standard, with its absorbance used to normalize the yeast expressed scFv-Fc activity. All the experiments were done in triplicate and standard deviation was calculated.

### Sandwich Assay

To determine detection limits, a sandwich assay was performed using yeast displayed antibodies and scFv-Fc fusions expressed from yeast. Eight of the anti-Ag85 scFv tested in ELISA were cloned into the yeast display vector pDNL6 and transformed into *S. cerevisiae* EBY100. Single clones were induced and resuspended in serial Ag85 dilutions (ranging from 5–100 nM). After incubation and washing, the yeast cells, with bound Ag85 attached, were resuspended in culture supernatants containing secreted scFv-Fc fusions. After incubation and washing, cells were resuspended in anti-rabbit-Alexa-633 (Invitrogen, Carslbad) to detect soluble scFv-Fc bound to Ag85. scFv-Fc fusions recognizing bound Ag85 at a site different to the yeast displayed scFv will be able to bind the Ag85 and confer a positive signal to the yeast. After washing and resuspension in PBS, fluorescence data were collected using the FACSAria and analyzed using DIVA software. The yeast population was gated and the mean fluorescent values for allophycocyanin (APC) (corresponding to the binding of scFv-Fc to antigen immobilized by the yeast displayed antibodies) were recorded using appropriate excitation lasers and emission filters.

The same assay was used to test the detection of Ag85 in human serum, except that antigen was serially diluted in 1∶50 serum diluted in PBS.

## Results

### Selection of Ag85-binding Clones from a Naïve Human Library

Figure 1 describes our strategy to select scFvs against Ag85 using phage and yeast display. Phage display is used to preselect the vast diversity of our naïve phage antibody library to a diversity compatible with subsequent analysis and selection by yeast display. After two rounds of phage selection, diversity was reduced from ∼10^11^ different scFv [22] to 70,000 colony forming units. This was cloned into our yeast display vector by gap repair [21] with a greater than ten fold coverage of the phage scFv output, ensuring maintenance of diversity. 22.2% of yeast displayed a functional scFv, of which 10% (2.1% of total population) bound Ag85, and less than 0.5% (0.1% of total) showed non-specific binding to streptavidin (data not shown).

### Yeast Display Mini-library Sorting

Yeast displaying Ag85 binding scFvs were sorted by incubating 2×10^6^ induced yeast mini-library cells with 100 nM of biotinylated Ag85, streptavidin-AlexaFluor-633 and phycoerythrin labeled anti-SV5 to measure scFv display levels. The top 1% of Ag85 binding yeast was sorted, using the P4 gate in Figure 2A, grown in selective media and regrown in induction media prior to reanalysis and repeat sorting (Figure 2B). After one sort, the percentage of yeast displaying scFvs recognizing Ag85 reached 25.1%. After sorting the top 1% of this population (sort gate P5 in Figure 2B) the percentage of Ag85 binding cells increased to 35.5% (Figure 2C), while the percentage of streptavidin binding clones remained constant at 3.2% for both (Figure 2D–E).

### Monoclonal Antibodies Displayed on Yeast

Sequencing of 192 random clones after each of the two rounds of flow sorting (96 each) revealed a total of 111 different scFvs (Table 1): 96 unique clones to either one or two rounds of yeast sorting and 15 common to both. Forty-eight of these, including the 15 shared ones, and an additional 33 found more than once in the sequencing, were tested for binding to Ag85 and two non-related proteins (Ubiquitin and Myoglobin). As shown in Figure 3, the mean fluorescent signal for each of these was clearly specific for Ag85, with very low recognition of the controls, indicating that at least 48 and probably more than 100 different specific antibodies against Ag85 have been isolated by this combined phage/yeast display approach.

### Antibody Response to Ag85 Complex and its Components

All the enrichment steps (phage selections and yeast display sorting) were performed using the Ag85 complex as target. However, as described above, Ag85 is composed of three subunits, Ag85A, B, C, which have 71–77% homology. For this reason we decided to use yeast display to test the antibodies for their ability to recognize the individual Ag85 components. Binding activity to the complete Ag85 complex was used to normalize the recognition of the 48 different scFvs for the three different components. None of the clones analyzed showed a strictly subunit specific binding activity, with a majority recognizing each of the subunits equally well (Figure 4). However, there were interesting patterns in which one or two of the subunits were clearly better recognized. For example, two clones (c2 and b11) showed a preference for Ag85A and C over Ag85B, while b7 recognized Ag85 B and C, and showed no binding to Ag85A. Other clones (e.g. a7, a12, b3, c7 c12 and d8) showed a preference for Ag85B. No clones preferentially recognizing Ag85A or Ag85C were isolated. The clones indicated with arrows were taken forwards for affinity analysis.

### Affinity

The affinities of the fourteen most interesting clones (in terms of binding profile during the screening and for their activity against the different Ag85 components – arrowed in Figure 4) were calculated by incubating each yeast-displayed scFv with varying quantities of Ag85 complex [25]. The binding constants for the scFv clones ranged from 22.1 nM to 432 nM with an average affinity of 126.1 nM, very similar to the 100 nM concentration of Ag85 used for yeast-based selection. The affinities of the antibodies obtained after one round of sorting had a broader range (39–432 nM, mean 180 nM) than those after two rounds of sorting (59.9–160 nM, mean 120.4 nM) or those found in common (22.1–110 nM, mean 81.8 nM) (Table 2).

### ELISA

Having selected large numbers of different scFvs with different affinities and recognition specificities for the different components of the main antigens, we recloned 14 scFvs into a yeast expression vector as scFv-Fc fusions [28]. These are constructs in which the scFv is directly fused to the N terminus of the hinge region of an immunoglobulin Fc region, and have the advantage that they are far more stable than scFvs alone, and preserve the binding properties of the original scFv. Furthermore, by using an Fc region derived from rabbit, we are able to use standard immunochemical secondary reagents. This allowed us to test the ability of these selected scFvs to specifically recognize Ag85 directly adsorbed to plastic in an ELISA format using standard anti-rabbit antibodies as secondary reagents (Figure 5). As can be seen, 11 of the 14 antibodies bind to Ag85 when adsorbed to plastic, without recognizing ESAT6 and myoglobin, the negative controls. Given that all 14 recognized Ag85 by flow cytometry, it is likely that the three negative antibodies (A3, A7 and D3) recognized conformational epitopes that may have been lost by adsorption to plastic, as described for other proteins [29].

### Sandwich Detection of Ag85

On the basis of these results, the top eight clones were further tested in an 8×8 sandwich assay, in which yeast displaying all eight selected antibodies were used as Ag85 capture reagents, and tested with all eight scFv-Fc fusions as detection reagents, with detection assessed using fluorescent anti-rabbit secondary antibodies. Prior to carrying out the sandwich assays, the yeast displaying each of the eight antibodies were shown to bind fluorescently labeled Ag85 (data not shown), indicating their functionality as potential capture agents. Figure 6 shows the results of these 64 sandwich assays. The individual histograms show binding of the designated scFv-Fc fusion antibodies to individual yeast particles displaying the indicated antibodies in the presence of 100 nM Ag85. The X axis shows fluorescence (i.e. the amount of scFv-Fc bound per individual yeast particle), while the Y axis represents the yeast count at each fluorescence level. The yeast in the peak to the left of the vertical line, represent the non-displaying fraction of yeast clones, due to plasmid loss [30], while yeast to the right of the vertical line are fluorescent as a result of a positive sandwich assay. The results in Figure 6 show that approximately a third of antibody pairs (21 of 64) showed a clear shoulder (e.g. C10 display; A2 scFv-Fc) indicating a positive result in the sandwich assay. Interestingly, three of the antibodies (C2, C9 and C11) gave positive results when used simultaneously as both capture and detection reagent, reflecting the fact that Ag85 is a trimer and that these antibodies (as well as most of the others) are able to recognize more than one subunit (as shown in Figure 4). Antibodies A2, B12 and F1 did not appear to give positive signals for any of the scFv-Fc fusions when used as capture agents, even though they were functional when used in the reverse formats (e.g. compare C10 display and A2 scFv-Fc with A2 display and C10 scFv-Fc). The remaining five displayed antibodies (C2, C9, C10, C11 and E6) were tested with all the scFv-Fc fusions to establish detection limits. The results of the best pairs are illustrated in Figure 7A, showing detection down to 6.1 nM in the absence, and 22.7 nM, in the presence, of serum for the best pair (C9 display and A2 scFv-Fc expression) (Figure 7B).

### Stability of the scFv-Fc Fusion

In our hands, one of the main problems with the commercially available antibodies when used for Ag85 detection is their tendency to be unstable, resulting in either a loss of binding activity, or increased non-specific binding, even when stored as recommended (Mukundan H et al, Ttuberculosis, in press 2012). In order to determine whether in vitro selected scFv-Fc fusions are more stable, we tested the stability of two of the unpurified antibodies for a period of four weeks at 4°C. The binding activity of the antibodies was detected in ELISA, (Figure 8), and normalized with respect to binding on the first day. As seen in Figure 8, there is no significant variation in the signal during this period, showing the stability of these constructs without the need for a purification step.

## Discussion

The direct detection of Ag85 in patient serum has been proposed as an effective diagnostic for TB [14], [15]. This requires stable specific high affinity antibodies, which to date have been either polyclonal [15], [31] or monoclonal [14], [15], , obtained by immunization. More recently, methods to generate antibodies using in vitro methods, such as phage and yeast display, have been developed. These have significant advantages over traditional immunization, including the speed with which antibodies can be selected and the ability to screen many more clones for highly specific properties. More significantly, once selected, it is relatively straightforward to improve many antibody properties such as: a) affinity [33], [34]; b) specificity, [35], [36]; and c) expression levels [37], [38]. In this paper, we have combined the two most powerful in vitro antibody selection platforms (phage and yeast display) to select a large number of antibodies against Ag85. This panel comprises a far greater antibody variety than usually obtained when carrying out phage display alone, significantly increasing the number of available anti-Ag85 antibodies. We believe this is due to the more efficient harvesting of the available antibody diversity. Depending upon the efforts expended in creating them, naïve antibody libraries have enormous diversity (>10^9^ different clones) [20], [22], [39], [40]. Theoretical [41] and practical [42], [43] studies have shown that, depending upon the desired affinity, a library size of ∼10^7^ clones should yield approximately one specific antibody, and that the number of selected antibodies should increase proportionally with library size. However, this has not been the case with phage display, where, unless especially heroic efforts are made [44], only 5–30 different specific antibodies are usually selected from large libraries. This is likely due to additional biases occurring during phage display selection [45] that do not appear to occur in yeast display [20], probably because expression control is tighter.

This combination of technologies exploits the advantages of each of the two platforms. After carrying out one to two rounds of selection in phage, the diversity is reduced from 10^9–11^ to 10^5–6^. This is compatible with straightforward cloning into yeast, and exploits the tremendous flexibility and sensitivity of the use of flow cytometry in subsequent antibody selection. Whereas traditionally up to 384 different clones are tested by ELISA after phage display, when phage and yeast display are combined as described here, every phage selected clone is tested individually: analogous to carrying out up to one million ELISAs, with the ability to sort out those that are positive in real time. This represents a significant advantage over using either phage or yeast display alone. In the former, the full selected diversity cannot be tested, while in the latter, it is already challenging to screen the naïve 10^9^ clone yeast antibody library that is available [20], and significantly more so to access even larger initial diversities. By combining the display platforms, all potential positives in a phage library can be screened.

Using this approach we identified 111 different positive antibodies out of only 192 sequenced clones, and anticipate that the application of deep sequencing to the selection [46], [47], [48] will identify significantly more positive clones. Although we identified many different antibodies recognizing Ag85, we were unable to identify any with absolute specificity for one of the subunits over the others. In part, this is likely to be due to the way the selection was carried out: using a trimer of similar subunits the dominant epitopes will be those found in all three subunits. Future attempts to select subunit specific antibodies will involve selection against one subunit in an excess of the others.

The data presented here focus on Ag85, a soluble antigen. However, we believe this technology is not restricted to soluble protein antigens, but can also be applied to the isolation of antibodies against membrane proteins. Both phage [49] and yeast [50], [51] display have been used to isolate antibodies in whole-cell panning experiments, and more recently specific protocols have been developed that allow the selection of antibodies against either random, or specific, membrane proteins [52]. Combining these well described methods with the approach described here, should allow the straightforward selection of antibodies against many different transmembrane proteins.

From these original 111 identified positive antibodies, we were able to carry out a series of rapid screening assays leading to 7 antibody pairs able to detect Ag85 down to 6.1 nM in the absence of serum and 22.7 nM in its presence. One attractive feature of this combined technology is the large number of different preliminary assays that can be carried out without the need to express or purify the antibodies. In particular, yeast displayed antibodies can be considered to be bead immobilized antibodies that only need to be grown up and induced in order to determine their affinity, specificity and epitope binding.

The direct detection of Ag85 components in serum is regarded as one of the most promising direct antigen detection potential diagnostic tests for TB [14], [15], particularly because of the very high level of secretion of this target [17]. This work describes a broad new diverse panel of anti-Ag85 (human) monoclonal antibodies, with great potential for the study and diagnosis of TB. Although we were unable to select antibodies with absolute specificity for the different Ag85 subunits, a number showed relative specificity, which could be significantly improved if required by maturation. Similarly, we anticipate it will be straightforward to significantly improve the detection levels reported here, by improving the affinities of the best capture and detection antibodies by yeast display [33], with the goal of achieving the sensitivity and specificity recommended by the WHO [53].
